# Survival outcomes of neoadjuvant immunochemotherapy versus chemotherapy for locally advanced esophageal squamous cell carcinoma

**DOI:** 10.1007/s00432-024-05793-4

**Published:** 2024-05-17

**Authors:** Huilai Lv, Fan Zhang, Chao Huang, Shi Xu, Jiachen Li, Bokang Sun, Chunyue Gai, Zhao Liu, Mingbo Wang, Zhenhua Li, Ziqiang Tian

**Affiliations:** 1 Department of Thoracic Surgery, The Fourth Hospital of Hebei Medical University, NO.12, JianKang Road, Shijiazhuang, Hebei China; 2 Hebei Key Laboratory of Accurate Diagnosis and Comprehensive Treatment of Esophageal Cancer, Shijiazhuang, Hebei China

**Keywords:** Esophageal squamous cell carcinoma, Survival outcomes, Neoadjuvant chemotherapy, Neoadjuvant immunochemotherapy

## Abstract

**Purpose:**

Neoadjuvant chemotherapy (NCT) is the standard preoperative treatment for resectable locally advanced esophageal squamous cell carcinoma (ESCC). Some studies reported neoadjuvant immunochemotherapy (NICT) could improve pathological response with manageable safety. However, few studies have compared the efficacy and safety of NICT and NCT, especially survival outcomes. In this study, we compared the efficacy and safety of NICT and NCT after a median follow-up of 36.0 months.

**Methods:**

This was a retrospective study with a 1:1 propensity score matching (PSM). Locally advanced ESCC patients treated with neoadjuvant sintilimab plus chemotherapy or chemotherapy followed by esophagectomy were reviewed. The primary outcome was recurrence-free survival (RFS).

**Results:**

Forty-five patients were identified in each group by PSM. The pathological complete response (pCR) rate in NICT and NCT group were 28.9% and 8.9% (P = 0.02). The hazard ratio (HR) was 0.396 (95% CI 0.171–0.919, p = 0.025) for RFS and 0.377 (95% CI 0.145–0.981, p = 0.038) for overall survival (OS), 3-year RFS was 80.6% and 62.1%, 3-year OS was 86.2% and 68.1%. Patients with pCR, MPR or downstaging had better 3-year RFS and 3-year OS. The incidences of postoperative complications and treatment-related adverse events (TRAEs) were similar.

**Conclusion:**

This trial preliminarily shows that NICT improves pathological and survival outcomes over NCT for resectable locally advanced ESCC, with acceptable and manageable safety.

**Supplementary Information:**

The online version contains supplementary material available at 10.1007/s00432-024-05793-4.

## Introduction

Esophageal cancer (EC) is a common malignant tumor, which ranks the seventh leading cause of cancer incidence and fifth leading cause of cancer mortality in China (Sung et al. 2021; Chen et al. 2023a; Zheng et al. 2024). China accounts for about half of the burden of EC worldwide (Sung et al. 2021; Chen et al. 2023a; Zheng et al. 2024). It was estimated about 224,000 new cases and 187,500 new deaths of EC occurred in China in 2022 (Zheng et al. 2024). Esophageal squamous cell carcinoma (ESCC) is the predominant histological type, accounting about 86% of all cases (Zheng et al. 2024). For the locally advanced resectable ESCC, multidisciplinary comprehensive treatment of neoadjuvant chemotherapy (NCT) or neoadjuvant chemoradiotherapy (NCRT) followed by esophagectomy is an effective strategy, which was confirmed to improve the survival of patients compared to surgery alone (Medical Research Council Oesophageal Cancer Working Group. 2002; Allum et al. 2009; Ando et al. 2012; van et al. 2012; Yang et al. 2018, 2021; Eyck et al. 2021). NCRT improve local pathological response compared to NCT, but more safety concerns affected its clinical application (Kumagai et al. 2014; Chan et al. 2018; Wang et al. 2021a; Zhang et al. 2022a). Many studies have shown that the overall survival (OS) of NCRT was not significantly improved compared to NCT (Zhang et al. 2022a; Tang et al. 2023; Kato et al. 2022). Thus, the novel neoadjuvant strategy of locally advanced ESCC is urgent to explore. However, the long-term survival of NCT or NCRT followed by esophagectomy for ESCC is still not promising.

Programmed cell death protein 1 (PD-1) inhibitors combined with chemotherapy have demonstrated promising antitumor effects and become the first-line standard care of advanced esophageal and gastroesophageal junction (GEJ) carcinoma (Lu et al. 2022; Luo et al. 2021; Wang et al. 2022; Xu et al. 2023. Sun et al. 2021; Li et al. 2021; Doki et al. 2022; Song et al. 2023). Neoadjuvant immunochemotherapy (NICT) is also considered to have great prospects and caused extensive concern. Several studies have demonstrated that NICT produced a higher pathological complete response (pCR) rate ranging from 21.7% to 50% with manageable toxicity profile (Zhang et al. 2021; Yang et al. 2022; Liu et al. 2022a, b. Lv et al. 2022, 2023; Yan et al. 2022; Liu et al.2022a, b; Zhang et al. 2022b; Chen et al. 2023b; Zhang et al. 2023a, b; Yang et al 2023; Yin et al. 2022; Wang et al.^2023^; Shen et al. 2021). However, few studies have compared the efficacy and safety of NICT and NCT alone, especially survival outcomes. In this study, we compared the efficacy and safety of NICT and NCT followed by esophagectomy in patients with resectable locally advanced ESCC after a median follow-up of 36.0 months in a retrospective consecutive cohort.

## Methods

### Study design and patient selection

This is a retrospective consecutive cohort study with propensity score matching (PSM) to compared the efficacy and safety of NICT and NCT followed by esophagectomy in patients with locally advanced resectable ESCC. The study was approved by the Ethics Committee of the hospital. Consecutive locally advanced ESCC patients treated with NICT or NCT alone followed by esophagectomy at The Fourth Hospital of Hebei Medical University between July 2019 and October 2021 were reviewed. The inclusion criteria were: an age of 18 years or older, both sexes, histologically confirmed ESCC, clinically staged as II-IVA, treated with neoadjuvant sintilimab combined with chemotherapy (albumin-bound paclitaxel and nedaplatin) or chemotherapy alone followed by esophagectomy. Patients with unresectable tumors or receiving other antitumor treatments before esophagectomy were excluded. Diagnosis and clinical stage were determined by chest-abdominal contrast enhanced computed tomography scan and/or enhanced magnetic resonance imaging, endoscopic ultrasound, cervical ultrasound. Position emission tomography-computed tomography was performed if necessary.

### Treatment

The eligible patients received 2–4 cycles of neoadjuvant sintilimab (200 mg, I.V, D1, Q3W) combined with chemotherapy (albumin-bound paclitaxel 260 mg/m^2^ and nedaplatin 80 mg/m^2^, I.V, D1, Q3W) or chemotherapy alone. Radiographic responses and restaging were assessed every 2 cycles by the same imaging means of the baseline. All patients suitable for radical esophagectomy underwent McKeown esophagectomy. The esophagectomy was usually performed within 4–8 weeks after neoadjuvant treatment. Pathological examination was carried out by two experienced pathologists according to the standard protocols. The survival follow-up was conducted according to the latest clinical guidelines, every 3 months during the first 2 years, and then every 6 months.

### Outcomes

The primary outcome was recurrence-free survival (RFS). RFS was defined as the time from the date of neoadjuvant treatment to the first documentation of recurrence or death. The secondary outcomes included the pCR rate, major pathological response (MPR) rate, tumor downstaging rate, OS and safety. The pCR was defined as no evidence of residual tumor in the primary tumor and resected lymph nodes. The MPR was defined as less than10% residual tumor in the primary tumor. Tumor downstaging was defined as a decrease in T stage or/and N stage. OS was defined as the time from the date of neoadjuvant treatment to death from any cause.

### Statistical analysis

R software (version 4.0.0) and SPSS software (IBM SPSS Statistics, version 26.0) were used for all statistical analyses. The continuous variables were presented as median and range, the comparison between two groups used Mann–Whitney U test. The categorical variables were presented as number and percentage, the comparison between two groups used Chi-square test or Fisher’s exact test. The 95% confidence intervals (CI) of pCR, MPR and tumor downstaging rate with was calculated using the Clopper–Pearson exact method. Median follow-up time was estimated using reverse Kaplan–Meier method. RFS and OS and the corresponding 95% CI were estimated using the Kaplan–Meier method, and the comparison between two groups used a log-rank test. A 1:1 PSM (caliper = 0.01) was conducted between NICT group and NCT group to minimize the bias of confounding variables. The propensity score was estimated by logistic regression models with following confounding variables: age, sex, smoking history, drinking history, Eastern Cooperative Oncology Group (ECOG) performance status (PS) Score, tumor location, clinical TNM stage, clinical T stage, and clinical N stage. The effect of neoadjuvant treatment among subgroups according to baseline characteristics were estimated using Univariable and multivariable Cox regression models. All statistical testing is two-tailed and performed at the 5% significance level.

## Results

### Baseline characteristics

A total of 181 eligible locally advanced ESCC patients completed NICT or NCT and underwent esophagectomy between July 2019 and October 2021 were included. In these patients, 131 patients received NICT and 50 received NCT. The NICT group had more clinical T3 stage patients (P = 0.033) than the NCT group before PSM (Table 1). After a one-to-one PSM, the final analysis included 45 patients in the NICT group and 45 patients in the NCT group, respectively. The baseline clinical characteristics were well balanced after PSM between two groups (Table 1).

**Table 1:** Baseline Clinical Characteristics

	Before matching			After matching		
	NICT (N = 130)	NCT (N = 51)	*P* value	NICT (N = 45)	NCT (N = 45)	*P* value
Age (year)						
≤ 60	44 (33.8%)	14 (27.5%)	0.41	9 (20.0%)	14 (31.1%)	0.23
> 60	86 (66.2%)	37 (72.5%)		36 (80.0%)	31 (68.9%)	
Sex						
Male	93 (71.5%)	40 (78.4%)	0.35	34 (75.6%)	35 (77.8%)	0.80
Female	37 (28.5%)	11 (21.6%)		11 (24.4%)	10 (22.2%)	
Smoking history						
Yes	59 (45.4%)	27 (52.9%)	0.36	23 (51.1%)	23 (51.1%)	> 0.999
No	71 (54.6%)	24 (47.1%)		22 (48.9%)	22 (48.9%)	
Drinking history						
Yes	61 (46.9%)	29 (56.9%)	0.30	25 (55.6%)	26 (57.8%)	0.83
No	69 (53.1%)	22 (43.1%)		20 (44.4%)	19 (42.2%)	
ECOG PS score						
0	84 (64.6%)	36 (70.6%)	0.19	29 (64.4%)	31 (68.9%)	0.82
1	38 (29.2%)	15 (29.4%)		15 (33.3%)	14 (31.1%)	
2	8 (6.2%)	0 (0.0%)		1 (2.2%)	0 (0.0%)	
Tumor location			0.80			0.90
Upper	20 (15.4%)	6 (11.8%)		7 (15.6%)	6 (13.3%)	
Middle	61 (46.9%)	24 (47.1%)		19 (42.2%)	21 (46.7%)	
Lower	49 (37.7%)	21 (41.2%)		19 (42.2%)	18 (40.0%)	
Clinical TNM Stage			0.47			0.29
II	49 (37.7%)	22 (43.1%)		18 (40.0%)	17 (37.8%)	
III	73 (56.2%)	24 (47.1%)		26 (57.8%)	23 (51.1%)	
IVA	8 (6.2%)	5 (9.8%)		1 (2.2%)	5 (11.1%)	
Clinical T stage			0.03			0.13
T2	13 (10.0%)	12 (23.5%)		5 (11.1%)	6 (13.3%)	
T3	111 (85.4%)	35 (68.6%)		40 (88.9%)	35 (77.8%)	
T4a	6 (4.6%)	4 (7.8%)		0 (0.0%)	4 (8.9%)	
Clinical N stage			> 0.999			0.92
N0	42 (32.3%)	17 (33.3%)		16 (35.6%)	14 (31.1%)	
N1	68 (52.3%)	26 (51.0%)		21 (46.7%)	24 (53.3%)	
N2	18 (13.8%)	7 (13.7%)		7 (15.6%)	6 (13.3%)	
N3	2 (1.5%)	1 (2.0%)		1 (2.2%)	1 (2.2%)	

### Pathological and survival outcomes

All patients completed McKeown esophagectomy in the NICT and NCT group. The R0 resection rate in the NICT group was comparable to NCT group. The pCR rate, MPR rate and tumor downstaging rate in the NICT group were significantly higher than those NCT group both in the original cohort and PSM cohort. (Table 2). The median number of removed lymph nodes were similar in both groups, with 30 (range 20–64) in the NICT group and 27 (range 20–50) in the NCT group. The adjuvant treatment was decided by multidisciplinary team according to the efficacy and safety of neoadjuvant treatment, postoperative recovery and patient's informed willingness. In the NICT group, 21 (46.7%) patients received adjuvant therapy, including 17 (37.8%) patients receiving adjuvant immunochemotherapy, 2 (4.4%) patients receiving adjuvant immunotherapy, and 2 (4.4%) patient receiving adjuvant chemotherapy. The median cycle of immunotherapy was 7 (range, 1–17), the median cycle of chemotherapy was 2 (range, 1–4). In the NCT group, 17 (37.8%) patients received adjuvant chemotherapy, The median cycle of chemotherapy was 2 (range, 1–4).

**Table 2:** The Pathological Outcomes

	Before matching			After matching		
	NICT (N = 130)	NCT (N = 51)	*P* value	NICT (N = 45)	NCT (N = 45)	*P* value
R0 resection	130 (100%, 95% CI 97.2–100%)	50 (98.0%, 95% CI 89.6–100%)	0.28	45 (100%, 95% CI 92.1–100%)	45 (100%, 95% CI 92.1–100%)	> 0.999
pCR	37 (28.5%, 95% CI 20.9–37.0%)	4 (7.8%, 95% CI 2.2–18.9%)	0.003	13 (28.9%, 95% CI 16.4–44.3%)	4 (8.9%, 95% CI 2.5–21.2%)	0.02
MPR	67 (51.5%, 95% CI 42.6–60.4%)	11 (21.6%, 95% CI 11.3–35.3%)	< 0.001	25 (55.6%, 95% CI 40.0–70.4%)	10 (22.2%, 95% CI 11.2–37.1%)	0.001
Tumor downstaging	83 (63.8%, 95% CI 55.0–72.1%)	22 (43.1%, 95% CI 29.3–57.8%)	0.01	31 (68.9%, 95% CI 53.4–81.8%)	21 (46.7%, 95% CI 31.7–62.1%)	0.03

**Fig. 1:**
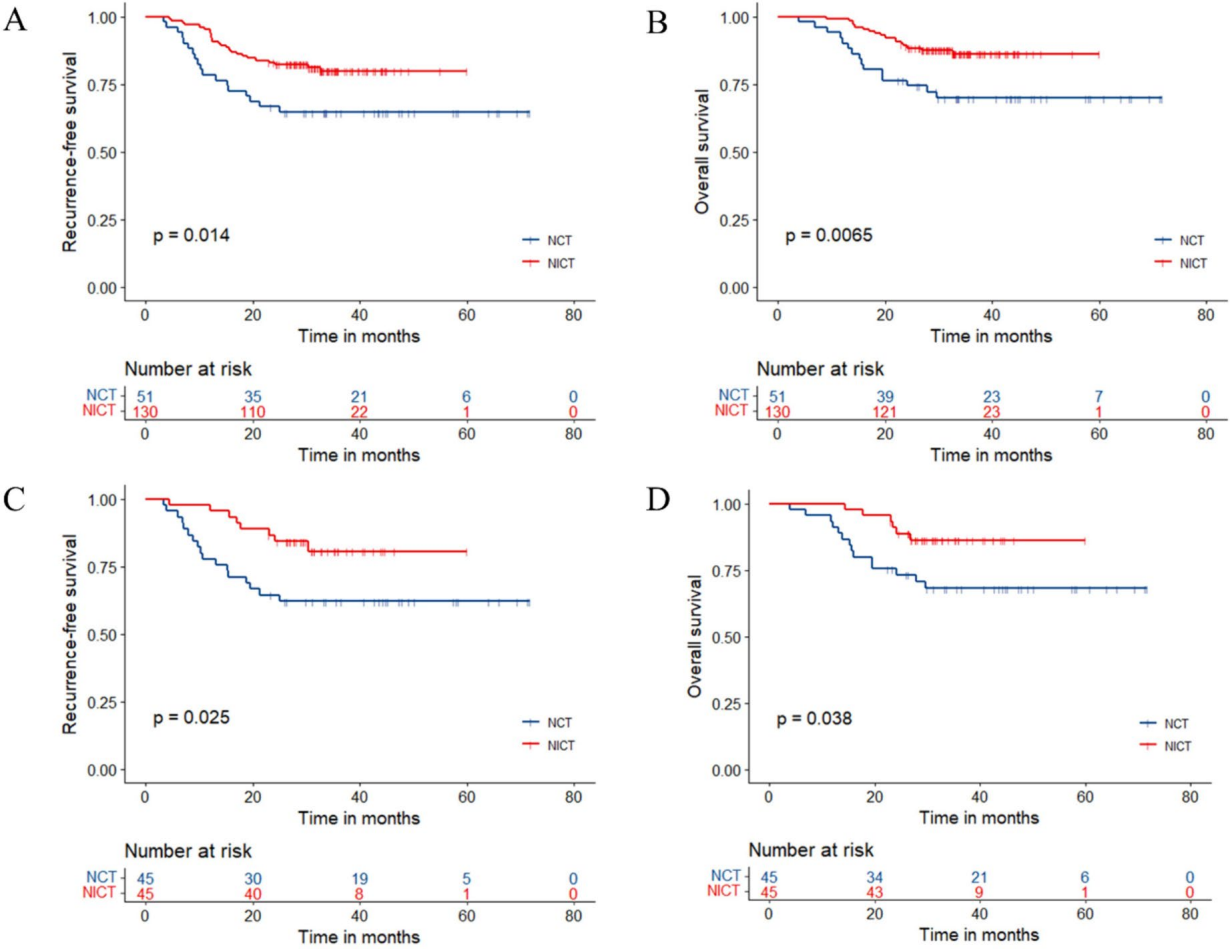
Kaplan–Meier estimates of RFS and OS. **A** RFS of the original cohort before matching. **B** OS of the original cohort before matching. **C** RFS of the PSM cohort after matching. **D** OS of the PSM cohort after matching

In the original cohort before matching, the median follow-up time was 34.3 (95% CI 33.2–35.7) months, with 45.0 (95% CI 40.8–50.3) months in the NCT group and 3.1 (95% CI 32.7–34.8) months in the NICT group. 25 (19.2%) patients of the NICT group and 18 (35.3%) patients of the NCT group experienced RFS events. The median RFS and median OS in both groups had not been reached yet (Fig. 1). The hazard ratio (HR) was 0.477 (95% CI 0.260–0.875, p = 0.014) for RFS and 0.394 (95% CI 0.197–0.789, p = 0.0065) for OS in the NICT group versus the NCT group. 2-year, 3-year RFS rate were 83.1% (95% CI 76.9–89.8%) and 79.8% (95% CI 72.9–87.4%) in the NICT group and 66.7% (95% CI 54.9–80.9%) and 64.6% (95% CI 52.8–79.2%) in the NCT group. 2-year, 3-year OS rate were 89.2% (95% CI 84.0–94.7%) and 86.3% (95% CI 80.3–92.7%) in the NICT group and 76.5% (95% CI 65.7–89.0%) and 70.0% (95% CI 58.3–84.0%) in the NCT group.

**Fig. 2:**
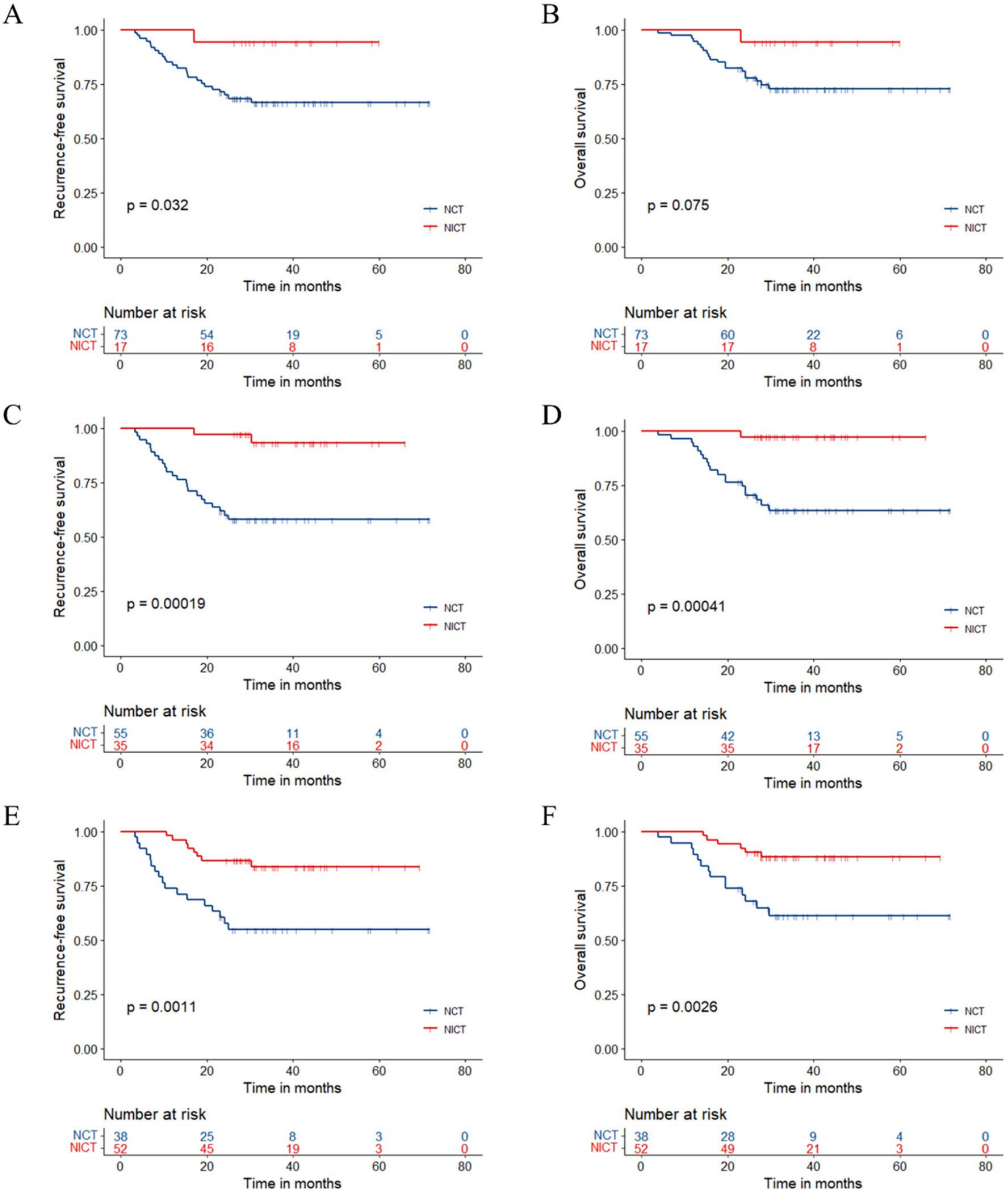
Kaplan–Meier estimates of RFS and OS stratified by pathological responses of the PSM cohort. **A** RFS of the pCR group and the non-PCR group. **B** OS of the pCR group and the non-PCR group. **C** RFS of the MPR group and the non-MPR group. **D** OS of the MPR group and the non-MPR group. **E** RFS of the tumor downstaging group and not achieving tumor downstaging group. **F** OS of the tumor downstaging group and not achieving tumor downstaging group

After PSM, the median follow-up time was 36.0 (95% CI 33.1–42.7) months, with 45.4 (95% CI 42.9–57.6) in the NCT group and 31.7 (95% CI 29.7–36.0) in the NICT group. 8 (17.8%) patients of the NICT group and 17 (37.8%) patients of the NCT group experienced RFS events. The median RFS and median OS in both groups had not been reached yet (Fig. 1). The hazard ratio (HR) was 0.396 (95% CI 0.171–0.919, p = 0.025) for RFS and 0.377 (95% CI 0.145–0.981, p = 0.038) for OS in the NICT group versus the NCT group. 2-year, 3-year RFS rate were 86.7% (95% CI 77.3–97.2%) and 80.6% (95% CI 68.9–94.1%) in the NICT group and 64.4% (95% CI 51.9–80.1%) and 62.1% (95% CI 49.5–78.1%) in the NCT group. 2-year, 3-year OS rate were 91.1% (95% CI 83.1–99.8%) and 86.2% (95% CI 76.6–97.1%) in the NICT group and 75.6% (95% CI 64.0–89.2%) and 68.1% (95% CI 55.6–83.5%) in the NCT group. The pCR, MPR, tumor down-staging patients have the significantly better survival outcomes (Table 3**, **Fig. 2). The univariable and multivariable Cox regression analysis identified the baseline factors including clinical N stage, ECOG PS score, neoadjuvant therapy regimen as independent predictors associated with RFS (Table 4).

**Table 3:** Comparisons Between Pathological Response Subgroups

PSM cohort (N = 90)	RFS HR (95% CI)/*P* value	3-year RFS (95% CI)	OS HR (95% CI)/*P* value	3-year OS (95% CI)
pCR (N = 17)	0.150 (0.020–1.112)	94.1% (83.6–100%)	0.194 (0.026–1.450)	94.1% (83.6–100%)
Non-pCR (N = 73)	*P* = 0.032	66.3% (56.1–78.4%)	*P* = 0.075	72.7% (62.8–84.1%)
MPR (N = 35)	0.105 (0.025–0.447)	93.1% (84.2–100%)	0.066 (0.009–0.495)	97.1% (91.8–100%)
Non-MPR (N = 55)	*P* < 0.001	57.9% (46.1–72.6%)	*P* < 0.001	63.4% (51.4–78.3%)
Downstaging (N = 52)	0.270 (0.117–0.627)	83.7% (73.7–94.9%)	0.256 (0.098–0.666)	88.1% (79.6–97.6%)
Non-Downstaging (N = 38)	*P* = 0.0011	54.8% (40.9–73.3%)	*P* = 0.0026	61.2% (47.0–79.7%)

**Table 4:** Univariable and multivariable Cox regression analysis according to baseline characteristics

Variable	Events, No./total No	Univariable analysis		Multivariable analysis*	
		HR (95% CI)	P value	HR (95% CI)	P value
Age					
≤ 60	8/23 (34.8%)				
> 60	17/67 (25.4%)	0.656 (0.283–1.521)	0.326		
Sex					
Male	21/69 (30.4%)				
Female	4/21 (19.0%)	0.558 (0.191–1.626)	0.285		
Tumor location					
Upper esophagus	2/13 (15.4%)				
Middle esophagus	13/40 (32.5%)	2.253 (0.508–9.986)	0.285		
Lower esophagus	10/37 (27.0%)	1.863 (0.408–8.504)	0.422		
Clinical T stage					
2	1/11 (9.1%)				
3	23/75 (30.7%)	3.872 (0.523–28.681)	0.185		
4a	1/4 (25.0%)	3.284 (0.205–52.520)	0.401		
Clinical N stage					
0	4/30 (13.3%)				
1	15/45 (33.3%)	2.943 (0.976–8.873)	0.055	4.448 (1.397–14.161)	0.012
2/3	6/15 (40.0%)	3.264 (0.921–11.571)	0.067	3.533 (0.981–12.719)	0.053
ECOG PS score					
0	10/60 (16.6%)				
1/2	15/30 (50.0%)	3.809 (1.707–8.499)	0.001	6.864 (2.747–17.150)	< 0.001
Neoadjuvant therapy					
NCT	17/45 (37.7%)				
NICT	8/45 (17.7%)	0.396 (0.171–0.919)	0.031	0.208 (0.081–0.529)	0.001

### Safety profile

The treatment-related adverse events (TRAEs) were comparable between two groups (Supplementary Table 1). In the PSM cohort, twenty (44.4%) and 6 (13.3%) patients of the NICT group developed grade 1–2 and grade 3–4 TRAEs, respectively. Twenty-one (46.7%) and 5 (11.1%) patients developed grade 1–2 and grade 3–4 TRAEs in the NCT group. However, the most common grade 3–4 TRAEs were neutropenia (6.7%, 6.7%), leukopenia (6.7%, 4.4%) in both groups.

No patients reported intraoperative complications. Postoperative complications were also comparable between both groups (Supplementary Table 2). In the PSM cohort, twenty-one (46.7%) and 21 (44.4%) patients developed grade 1–2 postoperative complications in the NICT group and NCT group, respectively. Two (4.4%) patients in the NICT group occurred grade 3–4 postoperative complications, with one patient had both grade 3 pulmonary infection and acute respiratory failure, and one had both grade 3 pulmonary infection. One (2.2%) patient in the NCT group had grade 4 pulmonary infection. No surgical mortality was reported in both groups.

## Discussion

This is the first 3-year follow-up outcomes of neoadjuvant immunochemotherapy versus chemotherapy for ESCC. The results preliminarily show sintilimab combined with chemotherapy have pathological and survival benefit comparable to chemotherapy alone, without increasing the postoperative complications.

To date, the majority of previous studies on NICT just released the pathological outcomes. The first phase 3 ESCORT-NEO study compared NICT with NCT alone in resectable locally advanced ESCC. An early look at the data showed the pCR rate was significantly higher in the camrelizumab plus chemotherapy arms (28.0% in albumin-bound paclitaxel and 15.4% in paclitaxel arm) compared with chemotherapy alone (4.7%) (Li et al. 2024). In our study, the pCR rate was 28.9% in the sintilimab combined with chemotherapy group and 8.9% in the chemotherapy group in the PSM cohort, which were consistent with the results of ESCORT-NEO study and showed pathological outcomes benefit of NICT compared with the NCT.

The follow-up data was not matured of ESCORT-NEO study. The other prospective studies that released survival outcomes were all single-arm studies and the follow-up period was relatively short. A phase 2 study showed that the 1-year DFS and OS of neoadjuvant sintilimab and chemotherapy were 68.3% and 90.8% after a median follow-up of 14.6 months (Zhang et al. 2022b). The KEEP-G 03 study showed that the 1-year DFS of neoadjuvant sintilimab and chemotherapy was 78.9% after a median follow-up of 17.3 months (Chen et al. 2023b). Two-year outcomes from phase 2 NICE study showed that the 2-year OS and RFS rates were 78.1% and 67.9% after a median follow-up of 27.4 months (Yang et al. 2024). A previous 1:1 PSM analysis shown that the 2-year DFS rates of the NICT group and in NCT groups were 80.7% and 63.8% (HR, 0.448, P = 0.046), the 2-year OS rates in the NICT group was 83.2% and 72.3% in the NCT group (HR, 0.564, P = 0.189) (Jing et al. 2022). In our study, after a followed-up time of 3 years, the results showed the 3-year RFS and OS rate of NICT were 80.6% and 86.2% compared to 62.1% and 68.1% of NCT. Overall, all these survival outcomes preliminarily showed the survival benefit when combined with PD-1 inhibitor and chemotherapy in the neoadjuvant setting.

Currently, NCRT is another important standard treatment choice for locally advanced ESCC based on the CROSS study and NEOCRTEC5010 study, the pCR rate was more than 40% (van et al. 2012; Yang et al. 2018, 2021; Eyck et al. 2021). However, the improvements in pathological response did not translate into survival benefit. The long-term survival results demonstrated no significant differences between the NCRT and NCT (Zhang et al. 2022a; Kato et al. 2022). The poor control of occult systemic metastasis was believed one of the top most reasons (Yang et al. 2021; Nakashima et al. 2018; Pasini et al. 2005; Shapiro et al. 2015). The CROSS and NEOCRTEC5010 study showed the decrease in distant progression of the NCRT was mainly during the first 24 months (Yang et al. 2021; Shapiro et al. 2015). An inverse probability of treatment weighting (IPTW) analysis showed NICT and NCRT had the comparable R0 resection rate and pCR rate. However, the patients received NICT exhibited a better prognosis than NCRT patients, the 3-year OS rates were 91.7% and 79.8% (P = 0.032) and the 3-year DFS rates were 87.4% and 72.8% (P = 0.039) (Yu et al. 2024). The DFS and OS rate of NICT in this study during the first 36 months increased numerically compared with NCRT in previous studies (Yang et al. 2021; Shapiro et al. 2015; Wang et al. 2021b; Yu et al. 2024). Moreover, the presence of the whole tumor allows more immunogenic cell death induced by chemotherapy and broader T cell response, establish systemic immune surveillance (Versluis et al. 2020; Emens et al. 2015; Topalian et al. 2020). On the other hand, NCRT might increase the risk of severe adverse events, the postoperative complications and mortality (Kumagai et al.^2014^), but no increase in postoperative complications and mortality were observed with NICT in this study. Therefore, NICT might be the more optimized clinical strategy and could achieve greater clinical benefits.

Further analysis revealed that the pCR, MPR, tumor downstaging patients have significantly better survival outcomes. The result from a single-arm prospective study also revealed patients who achieved MPR had improved DFS and OS (Wang et al. 2023). The results preliminarily indicate that pCR and MPR might be used as alternative survival indicators for NICT, which is consistent with previous findings in NCT and NCRT (Rizvi et al. 2014; Blum et al. 2017; CHIU et al. 2020).

There are several limitations in this study. First, this retrospective study could potentially lead to bias. We tried our best to improve the comparability through PSM method, but the sample size is limited. Therefore, these findings required further validation by prospective head-to-head comparison studies. Second, the use of adjuvant therapy in the groups may potentially affect the outcomes. Third, longer follow-up is necessary to validate the long-term benefits of NICT compared to NCT for locally advanced ESCC.

## Conclusions

This trial preliminarily shows that NICT followed by esophagectomy improves pathological and survival outcomes over NCT among patients with resectable locally advanced ESCC, with acceptable and manageable safety. Long term survival validation is still needed and prospective randomized or head-to-head comparison studies are warranted.

### Supplementary Information

Below is the link to the electronic supplementary material.

Supplementary file1 (DOCX 52 KB)

## Data Availability

The datasets generated during and/or analysed during the current study are available from the corresponding author on reasonable request.
