# Supplementary material for: Survival outcomes of neoadjuvant immunochemotherapy versus chemotherapy for locally advanced esophageal squamous cell carcinoma
Source: J Cancer Res Clin Oncol. 2024 May 17;150(5):260. doi: 10.1007/s00432-024-05793-4 (PMC11101546; doi:10.1007/s00432-024-05793-4)
Supplement: Supplementary file 1 — Supplementary file1 (DOCX 52 KB) [file 432_2024_5793_MOESM1_ESM.docx]

**Survival outcomes of neoadjuvant** **immunochemotherapy versus** **chemotherapy for locally advanced** **esophageal squamous cell carcinoma**

Huilai Lv^1,2^, Fan Zhang^1,2^, Chao Huang^1,2^, Shi Xu^1,2^, Jiachen Li^1,2^, Bokang Sun^1,2^, Chunyue Gai^1,2^, Zhao Liu ^1,2^, Mingbo Wang^1,2^, Zhenhua Li^1,2^, Ziqiang Tian^1,2^

^1^Department of Thoracic Surgery, The Fourth Hospital of Hebei Medical University, Shijiazhuang, Hebei, China

^2^Hebei Key Laboratory of Accurate Diagnosis and Comprehensive Treatment of Esophageal Cancer, Shijiazhuang, Hebei, China

**Address correspondence to:** Ziqiang Tian. NO.12, JianKang Road, Shijiazhuang, Hebei, China E-mail: tianziqiang@hebmu.edu.cn**.** Fax: +86-311-66696452. Tel.: +86-18531118000

**Supplementary Table 1. Treatment-Related Adverse Events**

|  | Before matching | |  | After matching | |
| --- | --- | --- | --- | --- | --- |
|  | NICT (N=130) | NCT (N=51) |  | NICT (N=45) | NCT (N=45) |
| Any treatment-related adverse events | | |  | | |
| Grade 1-2 | 61（46.9%） | 25（49.0%） |  | 20（44.4%） | 21（46.7%） |
| Grade ≥3 | 17（13.1%） | 6（11.8%） |  | 6（13.3%） | 5（11.1%） |
| Leukopenia |  |  |  |  |  |
| Grade 1-2 | 32（24.6%） | 11（21.6%） |  | 11（24.4%） | 10（22.2%） |
| Grade ≥3 | 5（3.8%） | 3（5.9%） |  | 3（6.7%） | 2（4.4%） |
| Neutropenia |  |  |  |  |  |
| Grade 1-2 | 17（13.1%） | 6（11.8%） |  | 7（15.6%） | 5（11.1%） |
| Grade ≥3 | 11（8.5%） | 4（7.8%） |  | 3（6.7%） | 3（6.7%） |
| Thrombocytopenia |  |  |  |  |  |
| Grade 1-2 | 17（13.1%） | 5（9.8%） |  | 6（13.3%） | 4（8.9%） |
| Grade ≥3 | 2（1.5%） | 1（2.0%） |  | 1（2.2%） | 1（2.2%） |
| Anemia |  |  |  |  |  |
| Grade 1-2 | 15（11.5%） | 5（9.8%） |  | 4（8.9%） | 5（11.1%） |
| Grade ≥3 | 2（1.5%） | 2（3.9%） |  | 2（4.4%） | 1（2.2%） |
| Liver Abnormalities |  |  |  |  |  |
| Grade 1-2 | 12（9.2%） | 3（5.9%） |  | 4（8.9%） | 2（4.4%） |
| Grade ≥3 | 2（1.5%） | 1（2.0%） |  | 1（2.2%） | 0 |
| Alopecia |  |  |  |  |  |
| Grade 1-2 | 25（19.2%） | 10（19.6%） |  | 8（17.8%） | 9（20.0%） |
| Grade ≥3 | 0 | 0 |  | 0 | 0 |
| Sensory neuropathy |  |  |  |  |  |
| Grade 1-2 | 22（16.9%） | 10（19.6%） |  | 7（15.6%） | 7（15.6%） |
| Grade ≥3 | 1（0.8%） | 1（2.0%） |  | 0 | 1（2.2%） |
| Nausea |  |  |  |  |  |
| Grade 1-2 | 19（14.6%） | 9（17.6%） |  | 7（15.6%） | 7（15.6%） |
| Grade ≥3 | 2（1.5%） | 2（3.9%） |  | 2（4.4%） | 1（2.2%） |
| Vomiting |  |  |  |  |  |
| Grade 1-2 | 14（10.8%） | 5（9.8%） |  | 4（8.9%） | 5（11.1%） |
| Grade ≥3 | 2（1.5%） | 2（3.9%） |  | 2（4.4%） | 1（2.2%） |
| Hypothyroidism |  |  |  |  |  |
| Grade 1-2 | 12（9.2%） | 3（5.9%） |  | 4（8.9%） | 3（6.7%） |
| Grade ≥3 | 3（2.3%） | 1（2.0%） |  | 1（2.2%） | 1（2.2%） |
| Diarrhea |  |  |  |  |  |
| Grade 1-2 | 10（7.7%） | 3（5.9%） |  | 3（6.7%） | 3（6.7%） |
| Grade ≥3 | 0 | 0 |  | 0 | 0 |
| Fatigue |  |  |  |  |  |
| Grade 1-2 | 7（5.4%） | 2（3.9%） |  | 2（4.4%） | 1（2.2%） |
| Grade ≥3 | 0 | 0 |  | 0 | 0 |
| Immune-mediated colitis |  |  |  |  |  |
| Grade 1-2 | 3（2.3%） | 0 |  | 1（2.2%） | 0 |
| Grade ≥3 | 1（0.8%） | 0 |  | 1（2.2%） | 0 |
| Pneumonitis |  |  |  |  |  |
| Grade 1-2 | 2（1.5%） | 0 |  | 1（2.2%） | 0 |
| Grade ≥3 | 0 | 0 |  | 0 | 0 |
| Cardiotoxicity |  |  |  |  |  |
| Grade 1-2 | 1（0.8%） | 0 |  | 1（2.2%） | 0 |
| Grade ≥3 | 0 | 0 |  | 0 | 0 |

**Supplementary Table 2. Surgical Complications**

|  | **Before matching** | |  | **After matching** | |
| --- | --- | --- | --- | --- | --- |
|  | NICT (N=130) | NCT (N=51) |  | NICT (N=45) | NCT (N=45) |
| Any postoperative complications | |  |  |  |  |
| Grade 1-2 | 56(43.1%) | 21(41.2%) |  | 21(46.7%) | 20(44.4%) |
| Grade **≥3** | 4(3.1%) | 2(3.9%) |  | 2(4.4%) | 1(2.2%) |
| Pulmonary Infection |  |  |  |  |  |
| Grade 1-2 | 30(23.1%) | 12(23.5%) |  | 14(31.1%) | 11(24.4%) |
| Grade **≥3** | 3(2.3%) | 2(3.9%) |  | 2(4.4%) | 1(2.2%) |
| Arrhythmia |  |  |  |  |  |
| Grade 1-2 | 28(21.5%) | 10(19.6%) |  | 9(20.0%) | 9(20.0%) |
| Grade **≥3** | 1(0.8%) | 0 |  | 0 | 0 |
| Anastomotic Leakage |  |  |  |  |  |
| Grade 1-2 | 8(6.2%) | 3(5.9%) |  | 3(6.7%) | 2(4.4%) |
| Grade **≥3** | 0 | 0 |  | 0 | 0 |
| Acute Respiratory Failure |  |  |  |  |  |
| Grade 1-2 | 5(3.8%) | 2(3.9%) |  | 2(4.4%) | 2(4.4%) |
| Grade **≥3** | 1(0.8%) | 1(2.0%) |  | 1(2.2%) | 1(2.2%) |
